# No smoking gun: tobacco taxation and smuggling in Sierra Leone

**DOI:** 10.1136/tobaccocontrol-2021-057163

**Published:** 2022-06-02

**Authors:** Max Gallien, Giovanni Occhiali

**Affiliations:** Governance Cluster, Institute of Development Studies, Brighton, UK

**Keywords:** taxation, illegal tobacco products, public policy

## Abstract

**Objective:**

To evaluate the common industry claim that higher tobacco taxation leads to higher levels of smuggling, particularly in a limited state capacity setting.

**Design:**

This paper evaluates the effects of a tobacco tax increase in Sierra Leone on smuggling by using gap analyses. Its models are based on multiple rounds of the Demographic and Health Survey and customs data as well as newly collected data on cigarette prices.

**Results:**

The paper shows that despite a substantial increase in cigarette taxation, and despite the absence of other formal tobacco control policies, smuggling has not increased in Sierra Leone. Its primary model shows a decrease in cigarette smuggling by 16.74% following the tax increase, alongside a decrease in cigarette consumption more widely and an increase in tax revenue.

**Conclusions:**

By presenting a low income and lower enforcement capacity case study, this paper provides novel and critical evidence to the debate on the tax-smuggling link. Furthermore, it points to new questions on how states in these contexts can limit cigarette smuggling.

**WHAT IS ALREADY KNOWN ON THIS TOPIC**

- The claim that higher tobacco taxation leads to higher levels of smuggling, undermining tobacco control objectives, is a central part of tobacco industry discourse.
- This claim however has been subject to increasing empirical criticism in recent years, frequently highlighting that tobacco control policies can de-link taxation and smuggling.

**WHAT THIS STUDY ADDS**

- Due to methodological challenges in studying the relationship between smuggling and tax, there is a relative scarcity of good case studies.
- Critically, the empirical evidence base on this topic is limited in countries with lower state capacity, particularly in low-income countries and Africa.

**HOW THIS STUDY MIGHT AFFECT RESEARCH, PRACTICE AND/OR POLICY**

- This study expands the evidence base on the relationship between taxation and smuggling by providing an ideally positioned case study, new data and a result that further contributes to the rejection of the long-standing tobacco industry hypothesis that higher taxes lead to increased smuggling.
- Furthermore, this study suggests that higher tobacco taxation can also be an effective tool to decrease tobacco consumption and increase revenue in a lower state capacity context.

## Introduction

The early 21st century has seen a marked increase in tobacco control discussions on the African continent. While traditionally smoking has been less prevalent in Africa than in Europe or North America, smoking levels in Africa have increased substantially in recent years as they dropped in the global North.^1^ This has created a critical policy window for African governments to reduce public health damages in coming decades.^2^ Among tobacco control strategies, taxation is usually presented as the single most effective policy tool to decrease smoking and fund public health responses. Tobacco taxation is also frequently pointed to as a particularly effective strategy for low-income and middle-income countries as it is comparatively cheap to implement.^3^ Article 6 of the WHO Framework Convention on Tobacco Control (FCTC) explicitly recommends introducing price and tax measures, and WHO has suggested a benchmark for the total share of taxes of at least 75% of retail prices. As of 2019, only three African countries met that benchmark.^4^

Recent work has linked discrepancies between tobacco tax recommendations and their actual rates to lobbying by the tobacco industry.^5^ One central argument from the tobacco industry has been that higher taxes will lead to more smuggling, consequently lowering revenue, increasing crime and worsening health outcomes.^5–8^ The veracity of this claim, especially in the context of low-income and middle-income countries, is a central debate in tobacco control scholarship. Research in recent years has strongly contested the assumption that higher taxation will necessarily lead to higher levels of smuggling.^9–13^ In particular, several studies have emphasised that the relationship between smuggling and taxation is mediated through various aspects of state enforcement capacity such as tax administration standards, track and trace systems, border control measures and effective revenue services.^11 14–17^ Here, it is notable that there has been comparatively less work on the relationship between tobacco taxation and smuggling in countries with comparatively limited state capacity.^9 11–13 18–20^

This paper seeks to contribute to this discussion by providing new evidence about Sierra Leone, a low-income and limited state capacity context. In 2018, it introduced a new excise tax on cigarettes of 30% of retail price, while also increasing import duties on all tobacco products. Our study asks whether the increase in tobacco taxation in Sierra Leone has led to an increase in tobacco smuggling. It employs gap analysis and data from multiple iterations of the Demographic and Health Survey (DHS), customs data as well as newly collected data on cigarette prices to estimate the effects of the tax increase. It finds strong evidence that smuggling has not increased, and in fact likely decreased. It also finds that the tax increase has been associated with decreased levels in smoking and increased public revenues.

Sierra Leone provides a particularly fitting case study to examine the effects of tax increases on smuggling. If the relationship between tobacco taxation and smuggling was to be strong and positive, as frequently claimed by the tobacco industry, we would expect Sierra Leone to be a prime example for this dynamic.

Tobacco consumption in Sierra Leone is highly skewed towards cigarettes^21^ and has had substantial effects on health and public finances in the country—according to estimations in a United Nations Development Programme report, tobacco consumption is responsible for over 3300 deaths annually and imposes annual costs on the country equivalent to 1.5% of its GDP.^22^ Notably, it has no domestic tobacco production—all domestically consumed cigarettes are imported.

While Sierra Leone ratified the FCTC in 2009, there has been little new tobacco control legislation since. To date, it has not introduced bans on advertisement, information campaign, plain packaging or warning label regulations. Notably, it has introduced no tax stamps or track and trace system that could limit smuggling. However, through 2017 to 2018, Sierra Leone enacted a substantial tobacco tax reform, introducing a 30% ad valorem excise tax on the wholesale price of cigarettes, and raising import duty on all tobacco products twice from 10% to 35% of cost, insurance and freight import value (Finance Amendment Act 2018, Supplement to the Sierra Leone Extraordinary Gazette Vol. CXLIX, No. 64 dated 19 July 2018; The Finance Act, 2018, Supplement to the Sierra Leone Gazette Vol. CXLIX, No. 31 dated 1 March 2018; The Finance Act, 2017, Supplement to the Sierra Leone Gazette Vol. CXLVIII, No. 30 dated 8 June 2017). While substantially higher than the previous level, these rates still fall below those mandated by the 2017 Economic Community of Western African States directive.^23^

Sierra Leone shares large borders with Guinea and Liberia. There are indications that illicit trade is prevalent in the region. Evidence of cigarettes smuggling in Sierra Leone exists going back to the 1980s, highlighting involvement of the tobacco industry^24^ as well as the existence of smuggling routes from neighbouring Guinea.^25^ Anecdotal evidence suggests the existence of various brands and packets on the Sierra Leonean market that were not produced for sale in the country. There are strong indications that state capacity in Sierra Leone to combat smuggling is limited. The Global Initiative against Transnational Organised Crime index category on state resilience to organised crime activities lists Sierra Leone in 12th place out of 15 West African countries, noting high levels of corruption and underfunded institutions.^26^ No tax stamps or track and trace policies have been implemented before or during the timeframe studied in this paper.

The combination of a tax increase isolated from other tobacco control policies, the presence of smuggling networks and limited state enforcement capacity all make Sierra Leone a particularly fitting case study to test the relationship between smuggling and taxation. If the positive relationship between cigarette taxation and smuggling were to hold more generally, these are strong reasons to believe that it should be visible in Sierra Leone. If the substantive tax increase in Sierra Leone did not lead to a substantive increase in smuggling, this leaves much to be explained for a price-based approach to modelling smuggling.

## Method

This paper uses gap analyses to estimate changes in cigarette smuggling. Gap analysis quantifies illegal markets size as the difference between estimated total consumption and legal production or import. Used systematically and extensively by the UK,^27^ gap analysis has seen application in a variety of global contexts.^28 29^ Our analysis is particularly indebted to its detailed applications in South Africa in recent years.^9 11 30^

Gap analysis is based on a simple model defining total cigarettes consumption (Q) as the sum of legally (QL) and illegally (QI) consumed cigarettes:

Q=QL+QI

As QI cannot be directly observed, it is estimated as the gap between Q and QL.^30^ Q is normally given by the product of smoking intensity and smoking prevalence—with estimates produced through surveys—and QL is obtained through data provided by government agencies, such as customs services or revenue authorities. The methodological challenges of gap analyses often lie in obtaining the relevant data and in the assumptions that might be required, as discussed in the following paragraphs of this section.

As our interest is primarily in the effect of the change in tobacco taxation on the levels of smuggling, our focus is directed to the change in QI over time, rather than the overall level of QI at any one point. This somewhat facilitates our tasks, as the assumptions that we make on factors such as consumption under-reporting or smoking by the elderly are only required to be time-invariant to correctly estimate the direction of the change.

The key variables required for our gap analysis are the smoking prevalence and intensity within Sierra Leone’s population (to estimate total consumption), as well as the total value and unitary price of imported cigarettes (to estimate legal consumption as Sierra Leone as no domestic production is present).

We obtain smoking prevalence and smoking intensity from the DHS 2013 and 2019, which contains the percentage of smokers in the population and the number of cigarettes smoked in the previous 24 hours. Two main challenges emerged here.

First, 2019 respondents were asked how frequently they smoked (‘daily’ or ‘less than daily’), and data about smoking intensity was only collected for daily smokers. This complicates a direct comparison of the two rounds, as there is no information about frequency of smoking behaviour for 2013, only on the number of cigarettes smoked in the previous 24 hours for all those who smoke. Our main model tackles this by assuming that non-daily smokers smoke one cigarette every other day. As outlined in online supplemental appendix 1, we also run alternative models under a different assumption to check the robustness of our analysis.

10.1136/tobaccocontrol-2021-057163.supp1 Supplementary data

A second challenge is the fact the DHS survey only covers females between the age of 15 and 49 years and males between the age of 15 and 59 years. While these represent the majority of the population, ‘senior citizens’ likely also consume tobacco. Therefore, we apply the gendered distributions of smoking incidence and intensity obtained from the two DHS waves to all Sierra Leonean aged above 15 years. The procedure to obtain updated smoking population figures, as well as the check that these are not driving our results, are presented in online supplemental appendix 1.

Furthermore, the DHS might underestimate total consumption due to under-reporting. Recent studies typically account for this by inflating reported consumption by a fixed amount.^11^ While this is less relevant for our analysis if we assume that under-reporting is time invariant, we inflate consumption in our ‘main model’ by 20%, a value within the bounds of those typical in the literature.^2 11^ We include different inflation levels as controls in online supplemental appendix 1.

The total value of imported cigarettes was obtained from custom data made available by the National Revenue Authority (NRA) of Sierra Leone. While this was transaction-level data, missing information about quantities made the recovery of unitary price from this source impossible. Fortunately, the NRA implemented a country-wide survey on tobacco prices prior to the 2017 tax increase, which was also made available. In theory, by deflating the average wholesalers’ price to 2013 values with the tobacco-specific component of inflation, we could recover the relevant unitary price. However, the 2017 survey did not specify whether the reported price was pretax, and cigarettes were subject to an import duty of 20%, as well as import value-added tax of 15% and an environmental charge of 2.7%. Based on the values reported, we assume that prices in the survey include these taxes. However, results for the alternative assumptions that reported prices were pretax are reported in online supplemental appendix 1.

The issue does not apply for 2019 prices, as these are obtained from a new wave of the price survey, implemented in 2021 (price levels for 2019 was also obtained by deflating the prices reported in the 2021 survey using the tobacco-specific component of inflation) by the authors in conjunction with the NRA, where pretax prices were explicitly asked.Both waves of the survey also included information for importers, so that we could use importer only averages. However, the coverage is very limited—only three and nine importers were covered in 2017 and 2019, respectively. Hence, we prefer to use prices for the whole sample, but we report results for importer only prices in online supplemental appendix 1.

## Results

Common positions that argue against the imposition of higher tobacco taxation on the basis of smuggling argue more broadly that tobacco tax increases will lead to an increase in smuggling, and, in the stronger version of that argument, that revenue gains that the tobacco tax increase could generate will be offset by increases in smuggling.

Our gap analysis finds strong evidence to reject both arguments for Sierra Leone, although under slightly differing assumptions. As the section above has discussed, our main model is built on a number of assumptions on the behaviour of non-daily smokers, the smoking habits of ‘senior citizens’, the level of misreporting and our price data. Based on what we consider our baseline model—which assume non-daily smokers consume a cigarette every other day, include ‘senior citizens’ and inflate reported consumption by 20%—our model rejects both statements. In fact, it shows that the ‘gap’ between total cigarette consumption and its legal import, a likely proxy for the level of smuggling, has decreased substantially, from 42.67% in 2013 to 25.92% in 2019. This represents a decrease in the market share of illicit cigarettes of 16.74% of the total market. At the same time, the price for cigarettes has increased substantially and tax revenues from cigarettes has more than doubled in real terms (while tax revenue collected through Goods and Service Tax has fallen due to lower imports, this has been made up by the higher rates of import duty and excise tax) Notably, and in line with the expected effects of an increase in tobacco taxation, the number of smokers has decreased substantially across genders despite an increase in the total population. Consequently, illegal and legal imports decreased between 2018 and 2019. Table 1 summarises these results. In the estimation of this model, the tobacco tax increase has achieved all its key goals and has not led to an increase in smuggling.

**Table 1:** Summary statistics for number of smokers and cigarette-tax revenue, gap sizes in baseline model, 2013–2019

	2013	2019
Number of smokers (male)	519 684	429 053
Number of smokers (female)	89 945	78 505
Total tax revenue from cigarettes in million in Sierra Leone	16 537	39 342*
Cigarette ‘Gap’ in per cent of the whole market	42.67	25.92

Source, authors calculation based on Sierra Leone Demographic and Health Survey 2013 and 2019 and NRA data.

*Deflated to 2013 terms for comparability. The procedure for obtaining both 2013 and 2019 tax revenue is reported in online supplemental appendix 1.

In order to test the robustness of our conclusions we have estimated a total of 40 models, in which we have varied all the assumptions outlined in the previous section. We provide a full discussion of these models in online supplemental appendix 1, and summarise these results in table 2. Across these 40 models, 36 find that the gap—and proxy for the level of smuggling—has decreased between 2013 and 2019. Only four models find that the gap has increased—and in all cases, the increase is small in comparison to the substantial tax increase, with a maximum of 3.51%. As discussed in online supplemental appendix 1, we do not consider these four models to be among the most plausible presented here, as they all require strong assumptions about high levels of smoking among non-daily smokers and about the reporting of prices. Notably, this wider set of models demonstrates that no single assumption outlined in the section above is driving the results of the above model—we need to vary at least two assumptions for the model to generate a gap increase.

**Table 2:** Summary statistics for model results across different assumptions

	Number of models	Average	Minimum	Maximum
Gap increase	4	2.5%	1.82%	3.51%
Gap decrease	36	−11.18%	−0.41%	−25.62%

Source, authors calculation based on Sierra Leone Demographic and Health Survey 2013 and 2019 and NRA data.

Consequently, while this wider set of models is not able to reject the possibility that smuggling has increased, it shows relatively little support for it, as even when selecting all assumptions in order to produce a higher ‘gap’, at most a comparatively marginal increase in smuggling is obtained. This wider set of models also serves as a comprehensive rejection of the idea that revenue gains from the tax increase could be significantly offset by increases in smuggling.

Naturally, there are limitations to these results. First, our analysis observes correlations of consumption and imports between two periods of time—this does not make a causal argument. However, we believe it provides ample evidence to doubt the broad causal argument inherent in many tobacco tax discussions. Second, the gap that we observe is not in itself an observation of the level of smuggling, but merely a proxy for it. For example domestic, production, legal or illegal, could distort this relationship. However, there are no reports of either in the country, so we have no reason to believe this might be a significant factor. Third, we cannot exclude that the gap exhibited a non-linear trend over the period—decreasing significantly between 2013 and 2018 and increasing again between 2018 and 2019. If this is the case, what we consider a decrease in the gap would instead represent an increase over an already materialised (and unobserved) decrease. However, this seems unlikely given that, after decreasing between 2013 and 2015 due to the Ebola epidemic, the volume of imported cigarettes grew on average of 9.8% per year between 2016 and 2018, exhibiting a positive year-on-year growth in each year, before then decrease by 31.9% between 2018 and 2019, following the tax increase and in line with the fall in consumption observed in the most recent DHS survey. For the gap to first decrease and then increase in response to the raise in tax rate, we would require cigarette consumption to have steadily decreased from 2013 onwards at an approximately constant rate, while also not responding to the change of tax rate. Given the absence of any tobacco control policy prior to the tax increase of late 2017, and the sharp decline in import volume following the second raise in 2018, this seems unlikely.

The magnitude of the import drop between 2018 and 2019 might seem large, but we do not find it surprising for three reasons. First, it reflects the substantial decrease in the overall number of smokers documented above. Second, the corresponding decrease in import value is substantially lower—13.9%. The higher decrease in import volume is connected with the strong inflationary pressure that the country faced in 2019,^31^ caused by a currency devaluation, and to the recently increased tax rate. Both of these factors contributed to increasing cigarette prices, and therefore the difference between the drop in value and in volume. Third, cigarette importers had ample time to stockpile ahead of the tax increase, which was first announced in November 2016^32^ but fully implemented only in July 2018. Stockpiling for future sales over this period would represent a rational economic strategy. Indeed, import volume increased by 8.1% between 2016 and 2017 and by 11.9% between 2017 and 2018, despite a decrease in consumption which was likely already ongoing. Seen in these contexts, the drop in volume is then not cause for concern.

## Conclusion and implications

Our results strongly suggest that all main goals of the tobacco tax increase have been achieved in Sierra Leone: prices have increased, tax revenue has increased and smoking has decreased. At the same time, we find strong evidence to suggest that, contrary to common arguments, smuggling has decreased. From this emerges a clear policy assessment: the revenue authority of Sierra Leone has made the right decision in increasing tobacco taxation and can consider increasing it further to the WHO recommended level without fear of its effect being eclipsed through smuggling. Given the porosity of the country’s border and the lack of additional tobacco control measures, the same conclusion is then very likely to apply to many revenue authorities in low-income countries.

At the same time, our results also point to a puzzle. Recent arguments that have sought to de-link tobacco taxation and smuggling have often emphasised the effect of state enforcement capacity and of other tobacco control interventions.^33^ However, as we have noted, state capacity in Sierra Leone is comparatively limited, especially in the context of combatting organised crime. Sierra Leone does not have a tax stamp or track and trace system, and no new tobacco control policies have been introduced in the past few years. To us, this does not detract from the importance of building enforcement capacity and introducing tobacco control policies in limited-capacity contexts. Instead, it suggests that tobacco taxation can make substantial gains even in contexts where other control measures are not fully established yet.

Our hypothesis is that the explanation for this result can be found in the structure and functioning of the cigarette smuggling market in Sierra Leone. An analysis of mirror statistics, customs data and conversations with relevant actors suggest to us that cigarette smuggling in Sierra Leone should not be imagined primarily as bootlegging and small-scale operations across uncontrolled land borders. Instead, there are some indications that a substantial part of the country’s cigarette smuggling could occur through formal points of entry, and consequently likely via formal sector actors. These observations suggest that in this case the relevant form of ‘state enforcement capacity’ then lie in the state’s ability to control large borders or supply chains, and is dependent on its interaction with a small number of large-scale actors, potentially tied to the formal sector. This relates to an emerging literature on informal regulation of smuggling^34^ and could explain the decrease in smuggling and how even a ‘limited state capacity’ context like Sierra Leone could achieve this.

These results suggest that there is significant scope for new research and new discussions on the relationship between tobacco control and smuggling, especially in limited-capacity contexts. This includes the role of formal sector actors, informal regulation, different forms of state capacity and the role of points of entries. An older discussion, meanwhile, ‘does tobacco taxation necessarily cause smuggling’, should finally be put to rest.

## Data Availability

Data are available on reasonable request.
