# Supplementary material for: No smoking gun: tobacco taxation and smuggling in Sierra Leone
Source: Tob Control. 2022 Jun 2;32(6):729–33. doi: 10.1136/tobaccocontrol-2021-057163 (PMC10646925; doi:10.1136/tobaccocontrol-2021-057163)
Supplement: Supplementary data [file tobaccocontrol-2021-057163supp001.pdf]

Appendix I: Models Construction, Specifications and Results.

A total of 40 different models were estimated for this study, in order to verify that our results are robust to variations in the assumptions needed for implementing the methodology. These assumptions are:

1. Inclusion of tax component in the reported price. This is especially relevant for the first wave of the survey, as the questionnaire did not specify if the required price was net of taxes. The 2017 wave of Tobacco price survey – which we use to recover 2013 prices – was implemented before the tobacco tax increase reform came into force, so that cigarettes were subjected to the same taxation as 2013. These included an import duty, equal to 10% of the CIF value, an import VAT charge of 15% of CIF value and an environmental tax equal to 2.7% of CIF value. The 2017 tax reform abrogated the environmental tax, increased the import duty tariff to 20% of and introduced an excise tax equal to 30% of the wholesale price, and the 2018 tax reform further raised the import duty level to 35%. As previously stated, the questionnaire of the 2017 wave of the tobacco price survey did not make a clear distinction between pre-tax and post-tax prices, so that we cannot be sure if the prices reported include the various charges that existed at the time. This is an issue, as in order to recover the true quantity of imported cigarettes we need to have pre-tax unitary prices - post-tax unitary prices are always higher than pre-tax ones, and this would lead us to recover a quantity of imported cigarettes lower than the actual one. As we cannot be sure, we calculate the gap for 2013 both assuming that the reported prices are pre-tax and assuming the they are post-tax, with latter always leading to higher imported volumes and, everything else equals, lower gaps. This is not an issue in the 2021 wave of the survey, as it was explicitly asked to respondents to report prices net of taxes if they were importers.
2. Construction of average cigarette price. Both rounds of the price survey include information about brand demand, as well as including several cigarettes importers. From this information, we construct three different price measures. One is a simple average of all cigarette prices

from wholesaler, one is a weighted average of wholesalers' prices, where high-demand branded are weighted at 50%, medium-demand brands at 30% and low demand brands at 20%. Finally, we also construct a simple average price using only information from importers. Simple average prices are higher than weighted average ones, with importer-only averages being the lowest. As above, the higher the price, the lower the quantity of imported cigarettes, the higher the gap – everything else equal.

3. Treatment of non-daily smokers. While the DHS 2013 did not contain information about smoking frequency, the 2019 round does, and information about smoking intensity is only widely available for daily smokers. This poses issues of comparability, so that non-daily smokers are always included in the estimates, although in two different ways. In the "floor" scenario, non-daily smokers only smoke one cigarette every other day, while in the "ceiling" scenario they consume half as many cigarettes as a daily smoker. That is, we apply the same distribution of daily cigarette consumption of daily smokers to non-daily ones, but with smoking levels reduced to a half. In the "floor" scenario, the number of cigarettes consumed is lower, so that, everything else equal, the gaps are also lower.
4. Inclusion of senior citizens. The Demographic and Health Survey (DHS) only covers male respondents aged between 15 to 59 and female respondents aged 15 to 49, as it mostly targets information about reproductive health. However, there are no reason to assume that senior citizens do not smoke, so that they should reasonably be included in the estimates. In order to do so, we obtained the distribution of the overall population by gender and age cohort from the Housing and Census Population 2015 and from the Sierra Leone Integrated Housing Survey 2018, both implemented by Statistics Sierra Leone. Both reports included population growth rates, which we assumed to be homogenous across age cohorts. With this assumption, we extrapolated the dimension of the missing population brackets, using the 2015 census to augment the DHS 2013 and the 2018 survey to augment the DHS 2019. After obtaining the gendered distribution of smoking intensities from the two DHS, we applied it to the newly obtained populations. That is, we assume that both smoking incidence and the distribution of smoking intensity for the male cohort of those aged 60-80+ and the female cohort of those

aged 50-80+ are the same of those of the male cohort aged 15-59 and the female cohort aged 15-49 respectively. As a consequence, everything else equal, excluding senior citizens lead to lower cigarette consumption, hence to lower gaps.

5. Under-reporting of cigarettes consumed. It is widely thought that self-reported levels of cigarette consumption are under-estimated by respondents, due to both recall bias and undesirability of the habit. We consequently proceed to inflate the reported level of consumption by 5% in certain scenarios and 20% in others. Inflating reported consumption increases the number of cigarettes smoked, hence increasing the size of the gap.

Given the above assumptions, we construct 4 sets of scenarios:

a) Exclusion of senior citizens, no inflation of reported consumption.
b) Inclusion of senior citizens, no inflation of reported consumption.
c) Exclusion of senior citizens, inflation of reported consumption by 5%
d) Inclusion of senior citizens, inflation of reported consumption by 20%.

In each of the above sets of scenarios, we then calculate the difference between the gap in 2013 and the gap in 2019 using all constructed average prices - simple average, weighted average, importers-only simple average - either including or excluding the tax component for each average price other than importer averages, which are assumed net of taxes. Furthermore, for each price combination, we calculate the difference in gaps over time for both the "floor" level of consumption of non-daily smokers (one cigarette very other day) and to the ceiling level (half the number of cigarettes consumed by daily smokers). Consequently, for each of the 4 scenarios the combination of assumptions gives rise to 10 diverse differences in gaps, namely:

i. Simple average – pre-tax price – "floor" consumption
ii. Simple average – pre-tax price – "ceiling" consumption
iii. Simple average – post-tax price – "floor" consumption
iv. Simple average – post-tax price – "ceiling" consumption
v. Weighted average – pre-tax price – "floor" consumption

vi. Weighted average – pre-tax price – “ceiling” consumption

vii. Weighted average – post-tax price – “floor” consumption

viii. Weighted average – post-tax price – “ceiling” consumption

ix. Imported average – pre-tax price – “floor” consumption

x. Imported average – pre-tax price – “ceiling” consumption

Summary figures for the key variables are reported in Table.A1 below. We consider our baseline model the one calculated using weighted average prices, post tax with “floor” consumption by non-daily smokers, including senior citizens and with inflated consumption at 20% (scenario d-vii), as this seems the most reasonable set of assumptions. Let us walk the readers through the calculation.

Table.A1 Summary statistics of key variables under different scenarios.

	2013	2019
Number of smokers, no senior	551,529	444,356
Number of smokers, senior included	609,629	507,558
Cigarettes smoked, no senior, no inflation (floor 2019)	1,919,641,832	1,058,560,298
Cigarettes smoked, no senior, no inflation (ceiling 2019)		1,127,618,370
Cigarettes smoked, no senior, 20% inflation (floor 2019)	2,303,570,198	1,270,272,358
Cigarettes smoked, no senior, 20% inflation (ceiling 2019)		1,353,142,044
Cigarettes smoked, senior, no inflation (floor 2019)	2,112,296,231	1,197,457,323
Cigarettes smoked, senior, no inflation (ceiling 2019)		1,277,670,149
Cigarettes smoked, senior, 20% inflation (floor 2019)	2,534,755,478	1,436,948,787
Cigarettes smoked, senior, 20% inflation (ceiling 2019)		1,533,204,179
Simple average price, import price	51.090	139.734
Simple average price, cleared custom price	39.543	93.468
Weighted average price, import price	50.570	139.731
Weighted average price, cleared custom price	39.141	93.466
Importer average price, import price	42.307	130.508
Importer average price, cleared custom price	32.745	87.296

Source: Authors elaboration on DHS 2013 and 2019 and tobacco prices surveys.

In 2013, the import data obtained from NRA gives us an overall CIF value of imported cigarettes of 56.63 billion Leones (Le). From the 2017 wave of tobacco price survey, the average unitary price of a cigarette at wholesale, weighted for the demand of different brands, is Le111.93, which, deflated using the tobacco component of the Consumer Price Index (CPI), is equal to Le50.57. Assuming that the respondents gave us prices which include the tax component, once we account for a 10% CIF

import duty, a cascading GST of 15% and an environmental tax of 2.7% CIF, we have a unitary pre-tax import price of Le39.14 per cigarette. Applying this to overall import value, we obtain a volume of import equal to 1.45 billion cigarettes, which is the size of the legal market (QL in the model presented in the main body of the article). From the 2013 wave of the DHS, augmented to include 'senior citizen', we have a smoking population 609,629, which consumed during the year 2.1 billion cigarettes. After accounting for potential under reporting by smokers and inflating the cigarettes consumed by 20%, we have an overall consumption equal to 2.52 billion cigarettes, which is the size of the actual market (Q in the model). By subtracting QL from Q we obtain QI (1.08 billion cigarettes), which is equal to 42.67% of the actual market (QI/Q).

The process to obtain QL for 2019 is specular, simply starting from the 2021 round of the tobacco price survey, while there is one key difference in obtaining Q – a further assumption is required for the treatment of non-daily smokers. After accounting for the inclusion of 'senior citizens', in 2019 there are 507,558 smokers, of whom 427,042 smoke daily and 80,516 only occasionally. In the 'floor' scenario, we assume that non-daily smokers consume one cigarette every other day, hence consuming 14.65 million cigarettes during the year, which, added to the 1.18 billion cigarettes consumed by daily smokers, give us an actual consumption of 1.2 billion cigarettes. As for 2013, we proceed to inflate consumption by 20%, which yields 1.43 billion cigarettes, the Q for 2019. Given a QL of 1.06 billion, QI is equal to 372.51 million cigarettes, or 25.92% of the actual market (QI/Q). The obtained difference in gap size between 2019 (25.92%) and 2013 (42.67%) is -16.74%, hence equivalent to a sizeable reduction in smuggling.

Out of 40 obtained scenarios, the only one leading to a positive gap difference between the two periods (i.e., an increase in smuggling) are scenarios a-x (+1.91%), scenario b-x (+3.51%), scenario c-x (+1.82%) and scenario d-x (+2.92%). Let us also show the calculation for scenario b-x, the one yielding the highest increase in smuggling. As before, we start from a CIF value of imported cigarettes equal to Le56.63 billion in 2013. However, rather than the weighted average unitary price amongst wholesalers as in the previous example, this time we use the average unitary price amongst direct importers. From the 2017 survey, this is equal to Le93.64, which deflated to 2013 prices

becomes Le42.31. Differently from before, this time we assume that this price is already net of taxes, and hence we proceed directly to obtain an import volume (QL) of 1.33 billion cigarettes. As before, we augment the DHS 2013 data by including ‘senior citizens’, but, differently from the previous scenario, we do not assume under reporting of consumption amongst smokers, so that cigarettes consumed (Q) remains equal to 2.1 billion. As a consequence, the size of the illegal market is now 774.49 million cigarettes (QI), or 36.83% of the actual market (QI/Q).

As before, the process to obtain QL for 2019 is specular, while to obtain Q we need a further assumption on the behaviour of non-daily smokers. Differently from the previous scenario though, this time we use the ‘ceiling’ rather than the ‘floor’ assumption for their consumption. That is, we assume that non-daily smokers smoke with half the intensity of daily smokers – the same amount per day, but only every other day. As a consequence, this time their annual consumption is equal to 94.87 million cigarettes, which, added to the same 1.18 billion cigarettes consumed by daily smokers as per the previous scenario, yields 1.28 billion cigarettes. As in this case we do not assume any underreporting, this is already the Q for the year. Given a QL of 762.32 million cigarettes – different from the one of the previous scenario as we now use average importer prices, assumed to be net of taxes, also for 2019 – the resulting gap is 515.35 million cigarettes (QI), or 40.34% of the market (QI/Q). This time, the difference between the gap size in 2019 (40.34%) and that in 2013 (36.83%) is equal to 3.51%, corresponding to a slight increase in smuggling amongst the two periods.

Looking again at what scenarios yield increase in smuggling (a/b/c/d-x), it is possible to notice that the only combination of assumptions leading to an increase in smuggling is the use of the importer-only average price with a high cigarette consumption of non-daily smokers. Varying either of these two assumptions – that is, using importer-only average prices but with “floor” consumption (a/b/c/d-ix), or using “ceiling” consumption with any price other than importers-only averages (a/b/c/d-ii/iv/vi/viii) – leads instead to a decrease in gap. Even in the 4 scenarios in which smuggling increase, the increases in revenue from higher taxation more than makes up for the loss due to higher smuggling. Hence, we can decisively reject the argument that the increase in revenue from higher

tobacco taxes will be offset by increases in smuggling, and while we cannot as strongly reject that increases in smuggling will take place, we find very little support for it.

The procedure to calculate the tax revenue reported in Table 1 is subjected to two assumptions - perfect enforcement (i.e., all the taxes due are collected) and no mark-up from wholesalers (i.e., the excise value is calculated on the import price and ignores the profit of wholesalers). While both of these assumptions are clearly unrealistic, they allow us to identify the effect of the tax reform in an ideal situation. The figures reported have been obtained from the CIF value of imported cigarettes communicated by the NRA as follows. In 2013, the total CIF value for imported cigarettes was Le56.63 billion. The import duty rate was 10% of CIF value, hence equal to Le5.66 billion, the cascading (i.e., applied to the sum of the CIF value and the import duty) GST rate is 15%, hence equal to Le9.35 billion, and the Environmental tax rate is 2.7% of the CIF value, hence equal to Le1.53 billion, for a total of Le16.54 billion. In 2019, the total CIF value of imported cigarettes was Le99.49 billion. The import duty rate was 35% of CIF value, hence equal to Le34.82 billion, the cascading GST rate is still at 15%, hence yielding Le20.15 billion, and the Environmental Tax has been repealed. However, there is now an ad-valorem excise of 30% of wholesale price, which we assume equal to the CIF value, hence yielding Le29.85 billion, for a total of Le84.81 billion. For comparability, we deflate this figure to 2013 value through the normal CPI, which yields a total revenue of Le39.34 billion.

Appendix II: Price Survey

There currently is no publicly available data on cigarettes' prices in Sierra Leone. The price data used in this article comes from 2 waves of a tobacco price survey implemented by the Sierra Leone National Revenue Authority (NRA), the first in 2017 - with support from the World Bank - and the second in 2021 – with support from the International Centre for Tax and Development. The samples for the surveys were obtained from the taxpayers' registry of the NRA, while additional informal cigarette retailers were also included during the enumeration to obtain a better picture of actual prices.

In addition to price information, the surveys collected a variety of other information, including market demand for different brands, impact of inflation on cigarettes' prices, average price mark-up applied by different business, as well as different questions about each respondent's perception about market structure and impact of taxes on cigarette price and demand.

The first round of the survey targeted 123 respondents, of which 13 (10.6%) were wholesalers and 3 (2.4%) were direct importers. The majority of the respondents (46.3%) were located in Freetown, the capital city, followed by respondents in Bo (18.7%), Kenema (18.7%) and Makeni (16.3%), respectively the second, fourth and third cities in the country for population. Overall, the survey included 443 data points on cigarettes' prices, of which 56 (12.6%) were from wholesalers and 7 (1.6%) were from importers. The second round of the survey targeted 183 respondents, of which 38 (20.8%) were wholesalers and 9 were importers (4.9%). As for the first wave, the majority of respondents were located in Freetown (54.1%), followed by Kenema (18.6%), Makeni (15.3%) and Bo (12%). The survey included 695 data points on cigarettes' prices, of which 129 (18.6%) were from wholesalers and 23 (3.3%) were from importers.

Apart from a slightly more extensive set of quantitative questions, the main difference between the two surveys lies in the wording of the question on the price of cigarettes. The first wave of the survey simply asked respondents to provide prices at which products were "sold to the public", without specifying if these should have been net of taxes or including them. The second round of the survey asked instead both the price at which products were acquired and the price at which products were sold, explicitly stating that if the respondent imported directly the price net of import duties and VAT was required. In this way, it was possible to acquire a clearer picture of price markups along the value chain, as well as acquiring the most representative prices to calculate the quantity of cigarettes imported.

We are currently working with our partners to make the data from this survey publicly available. In the meantime, for any enquiries please contact the authors.
